# Perspectives of Australian policy-makers on the potential benefits and risks of technologically enhanced communicable disease surveillance – a modified Delphi survey

**DOI:** 10.1186/s12961-019-0440-3

**Published:** 2019-04-04

**Authors:** Chris Degeling, Jane Johnson, Gwendolyn L. Gilbert

**Affiliations:** 1 Australian Centre for Health Engagement, Evidence and Values, School of Health and Society, Faculty of Social Science, University of Wollongong, Building 233.G05D, Wollongong, NSW 2500 Australia; 2 Sydney Health Ethics, Sydney School of Public Health, University of Sydney, Sydney, NSW Australia; 3 Centre for Infectious Diseases and Microbiology, Westmead Institute for Medical Research, Westmead, NSW Australia; 4 Marie Bashir Institute for Infectious Disease and Biosecurity, University of Sydney, Sydney, NSW Australia

**Keywords:** Australia, technological innovation, infectious disease research, public health surveillance, policy implementation, expert consultation

## Abstract

**Background:**

Event-based social media monitoring and pathogen whole genome sequencing (WGS) will enhance communicable disease surveillance research and systems. If linked electronically and scanned systematically, the information provided by these technologies could be mined to uncover new epidemiological patterns and associations much faster than traditional public health approaches. The benefits of earlier outbreak detection are significant, but implementation could be opposed in the absence of a social licence or if ethical and legal concerns are not addressed.

**Methods:**

A three-phase mixed-method Delphi survey with Australian policy-makers, health practitioners and lawyers (*n* = 44) was conducted to explore areas of consensus and disagreement over (1) key policy and practical issues raised by the introduction of novel communicable disease surveillance programmes; and (2) the most significant and likely risks from using social media content and WGS technologies in epidemiological research and outbreak investigations.

**Results:**

Panellists agreed that the integration of social media monitoring and WGS technologies into communicable disease surveillance systems raised significant issues, including impacts on personal privacy, medicolegal risks and the potential for unintended consequences. Notably, their concerns focused on how these technologies should be used, rather than how the data was collected. Panellists held that social media users should expect their posts to be monitored in the interests of public health, but using those platforms to contact identified individuals was controversial. The conditions of appropriate use of pathogen WGS in epidemiological research and investigations was also contentious. Key differences amongst participants included the necessity for consent before testing and data-linkage, thresholds for action, and the legal and ethical importance of harms to individuals and commercial entities. The erosion of public trust was seen as the most significant risk from the systematic use of these technologies.

**Conclusions:**

Enhancing communicable disease surveillance with social-media monitoring and pathogen WGS may cause controversy. The challenge is to determine and then codify how these technologies should be used such that the balance between individual risk and community benefit is widely accepted. Participants agreed that clear guidelines for appropriate use that address legal and ethical concerns need to be developed in consultation with relevant experts and the broader Australian public.

**Electronic supplementary material:**

The online version of this article (10.1186/s12961-019-0440-3) contains supplementary material, which is available to authorized users.

## Introduction

Significant outbreaks of infectious disease have impacts that extend beyond morbidity and mortality. Since they are often unanticipated and unpredictable, such outbreaks can cause fear, economic instability and social upheaval [1, 2]. Establishing and maintaining surveillance systems are the foundation of effective outbreak detection and public health response. Moreover, effective routine surveillance is central to the rational allocation of resources and addressing health inequalities, which cannot be tackled unless made visible. Because surveillance relies on the collection, collation and interpretation of large amounts of data, technological innovation has the potential to enhance the efficiency and accuracy of population-based communicable disease research and surveillance systems. For example, the increasing availability of pathogen whole genome sequencing (WGS) and the collection of passive data generated by internet and mobile phone use within a population, will provide new resources for communicable disease research and surveillance activities [3–5].

Methods for characterisation of microbes have become increasingly sophisticated over the last 20 years. WGS technologies can provide rapid and accurate information about which microbial species and strain type is causing an outbreak and the timing and direction of transmission. Incorporating this information into surveillance systems will permit more accurate biological risk prediction and faster outbreak identification and tracking [6, 7], but also reveal information about individuals that many people would consider to be private [8]. Concurrent with these developments in microbiological analysis, there has been a similar rate of technological innovation in data management. Using the tools provided by ‘Big Data’, syndromic surveillance systems can track and integrate online data collected for unrelated purposes that potentially reflect disease activity in the community such as social media posts and internet searches [9–13]. Integrating either or both of these new sources of information into communicable surveillance practices has the potential to greatly enhance current systems. If linked electronically and scanned systematically, pathogen WGS data and user-generated online information could be mined to uncover new epidemiological patterns and associations much faster than traditional public health approaches [14–16]. Incorporating these new technologies and novel sources of information into established communicable disease surveillance systems should improve our understanding of the rate and direction of disease transmission between individuals and within populations, provide earlier warning and more accurate monitoring of outbreaks, and reduce uncertainty and public fear during their early stages [8, 17].

The benefits of earlier outbreak detection and response are significant but, when there is no immediate threat, the routine use of a new technology to capture more detailed, specific personal health information could be perceived as intrusive and a threat to privacy, no matter how great the potential benefits for research and practice. When implementing such systems, sovereign states, health authorities and surveillance officers must consider the organisational, political, legislative, personal and ethical implications of surveillance [18–20]. However, there are also ethical and medico-legal risks in not using available information that could protect local and, potentially, international communities from serious disease outbreaks [21, 22]. Health authorities and researchers in Australia, and elsewhere, are beginning to explore the use of pathogen WGS and event-based social media monitoring, but many jurisdictions still lack policies and structures to support these technologies [5, 23, 24].

In this paper, we report the results of a modified Delphi survey involving policy-makers and experts with backgrounds relevant to the legal, ethical and epidemiological dimensions of technology-enhanced surveillance systems. This method is particularly useful for technological forecasting and the evaluation of complex problems where (1) the rate of socio-technical change exceeds that which can be managed by technocratic styles of governance; and/or (2) objective data (and models and relations dependent on this data) are insufficient to explain or predict social actions [25]. Participants were drawn from two broad groups, namely (1) an established policy community centred on communicable disease control and (2) an emerging issues network of individuals interested in the social, legal and security dimensions of technological change and innovation.

Policy communities and issues networks are at either end of a continuum characterised by differing levels of internal cohesiveness, state support and access to, and ability to regulate, shared resources [26]. Policy communities are stable, institutionally entrenched groups who share values and broad policy preferences, interact frequently and participate in relatively equal communications to produce lasting policy outcomes; conversely, issue networks include increasingly influential academic, industry and interest groups varying in levels of co-ordination and access to resources, who discuss, critique and generate ideas for policy initiatives in specific policy areas [27, 28]. This study is part of a larger project to develop guidance for policy-makers; its aim is to engage these two networks in a conversation about the social, ethical and legal implications of the use of new technologies (online data monitoring and WGS pathogen fingerprinting) in infectious disease research, control and prevention. In this Delphi survey we sought to (1) identify perceived barriers to the adoption and effective implementation of new technology for communicable disease research and surveillance; and (2) explore areas of consensus or disagreement about potential threats or conflicts of interest between individuals, commercial entities and the broader community associated with it.

## Methods

### Participants

A heterogeneous and geographically dispersed group of Australian-based policy-makers and experts in infectious diseases, epidemiology, food safety, health informatics systems, and health and technology law were invited to participate in this Delphi survey. Noting that the boundary between the roles of policy-makers and expert advisors can be nebulous, we defined ‘policy-makers’ as individuals who participate in policy processes to create, order and maintain rules and structures of action, and ‘experts’ as individuals with knowledge and experience of the law and/or public health practices, relevant to infectious disease control and prevention, technological innovation, or both [29, 30]. Sampling was purposive, to ensure representation of the relevant disciplines and types of actors. Potential participants were identified through institutional websites and researchers’ professional networks.

### Delphi processes

The rationale underpinning Delphi surveys is that consensus about contentious issues carries more weight than individual opinions [31]. Anonymous data are collected from individuals, collated and then re-presented to the group to elicit further responses [32]. In this study, we analysed data iteratively in parallel with data collection. Rather than force consensus, we employed a modified technique that allowed participants to explain their views. Participants were asked to assess and respond to the levels of consensus/disagreement that emerged from each round to provide greater insight into the potential benefits, harms and risks of using new technologies to enhance communicable disease surveillance. Participants who completed each round were invited to participate in the next, but were free to withdraw at any time. We used an online survey platform (*Limesurvey*).

In round 1, we asked participants to respond to three hypothetical scenarios (available in Additional file 1) describing the use of a new technology for the purposes of communicable disease surveillance in (1) social media/online, (2) hospital/workplace, or (3) commercial environments. Responses were analysed qualitatively and coded thematically by two authors (CD and JJ) using framework analysis, a deductive matrix-based qualitative research method for ordering and synthesising textual data, developed by the National Centre for Social Research (United Kingdom). Framework analysis methods are especially well suited to conducting applied and policy-relevant research [33, 34]. During rounds 2 and 3, participants’ comments, key arguments and levels of consensus from previous rounds were presented as quotations, bar charts and summaries of qualitative findings, taking care to weigh different opinions and arguments equally. Individual comments were de-identified.

Additional data and comments were collected, using Likert scales and free text responses. On completion of each round, participants’ Likert scores were tabulated and free text answers analysed qualitatively, as described. The final stage of analysis during preparation of this report drew on the knowledge and professional experience of the research team, which includes expertise in health social science, bioethics, Australian health law, infectious diseases, microbiological laboratory and data sciences, and health protection. This study was approved by the University of Sydney Human Research Ethics Committee (#2016/819).

## Results

### Participants

Email invitations were sent to 92 potential participants, of whom 44 (47%) from a range of relevant roles and disciplines responded (Additional file 2: Table S1). Invitations included an individualised link to the online survey, through which participant consent was obtained. As expected, the panel size gradually decreased as participants withdrew [35], but the balance between sectors and characteristics of participants remained substantially constant (Additional file 2: Table S1).

### Enhanced communicable disease surveillance in digital environments

In round 1, panellists answered a series of structured questions about a scenario describing the use of a new online event-based surveillance system that monitors social media (Twitter, Facebook, etc.) for indicators of emerging public health risks. In the scenario, the new system picks up a post on Facebook from Bob, a 25-year-old high school teacher from Sydney. His post suggests that he may have contracted highly pathogenic avian influenza on a recent holiday to Indonesia. Online discussion of symptoms and work absences also suggests that Bob might have spread the infection to several of his colleagues. Delphi participants proposed a number of responses to this scenario. While the mandate to respond to a potential public health risk was seen to be clear, Delphi panellists disagreed about how to do this and how to contact those most at risk (particularly Bob and his colleagues). After reading each other’s comments and suggestions on the merits and risks of different strategies for addressing this acute situation, in rounds 2 and 3 we asked the panel to nominate which approach to contacting Bob they thought was the most appropriate.

**Fig. 1:**
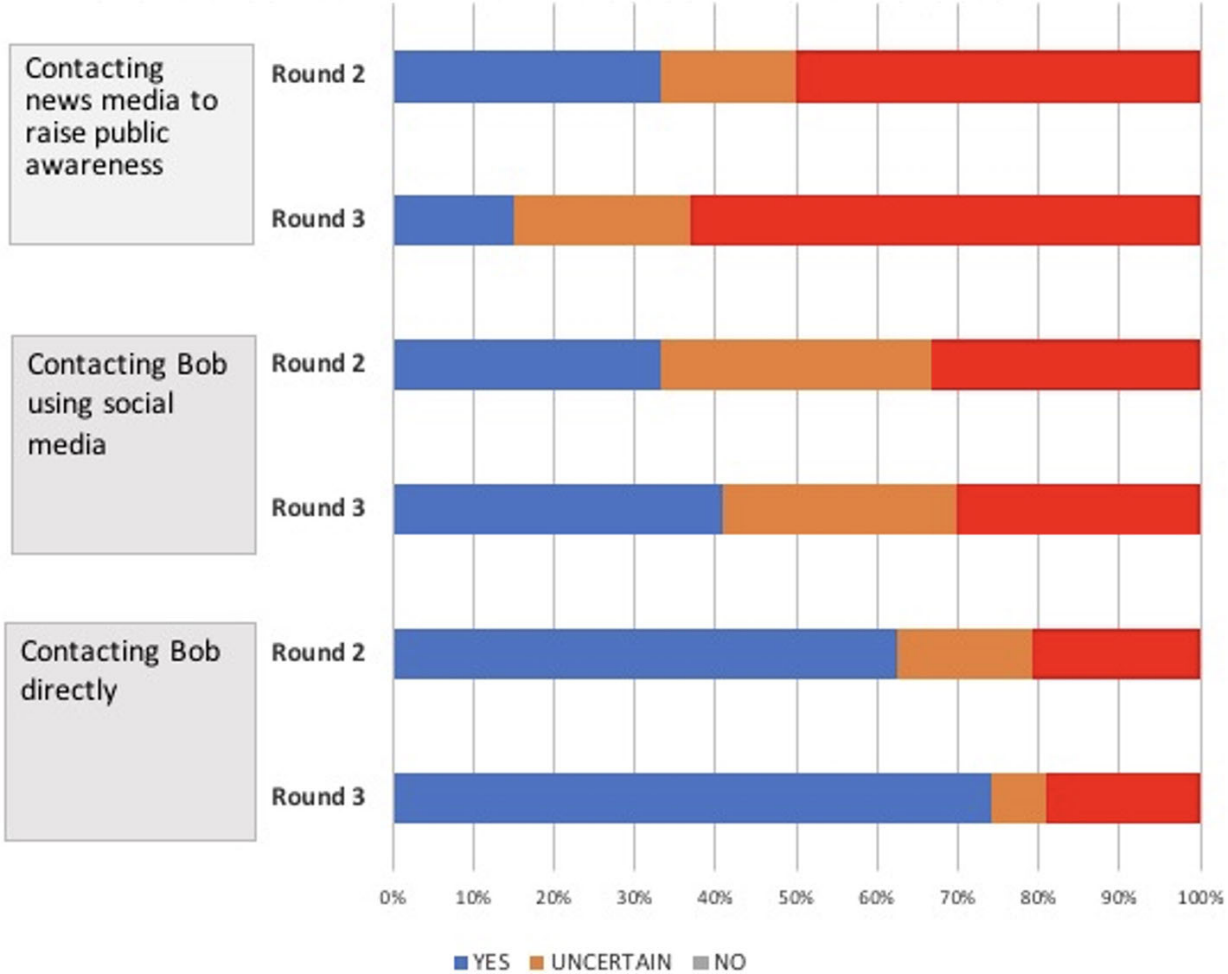
Panellist’s preferences as to how public health authorities should contact someone flagged by an online event-based communicable disease surveillance system

Figure 1 shows that, while a direct approach (speaking to Bob in person) was strongly favoured, a majority of panel members questioned the acceptability of using a social media platform for this communication, even though Bob had posted information about his health status in a public forum. Others were concerned that an online intervention could create panic by identifying Bob as a public health risk. As one panellist noted:

DP #12: “*Where individuals have already disclosed information themselves, then one can make a reasonable assertion that the information is therefore not private. This, however, is not the same as that individual allowing or being willing to have their social media platform used to contact or warn others or to make further enquiries, since all of these actions have the potential to alarm, stigmatise or otherwise negatively affect the social media user.*”

**Fig. 2:**
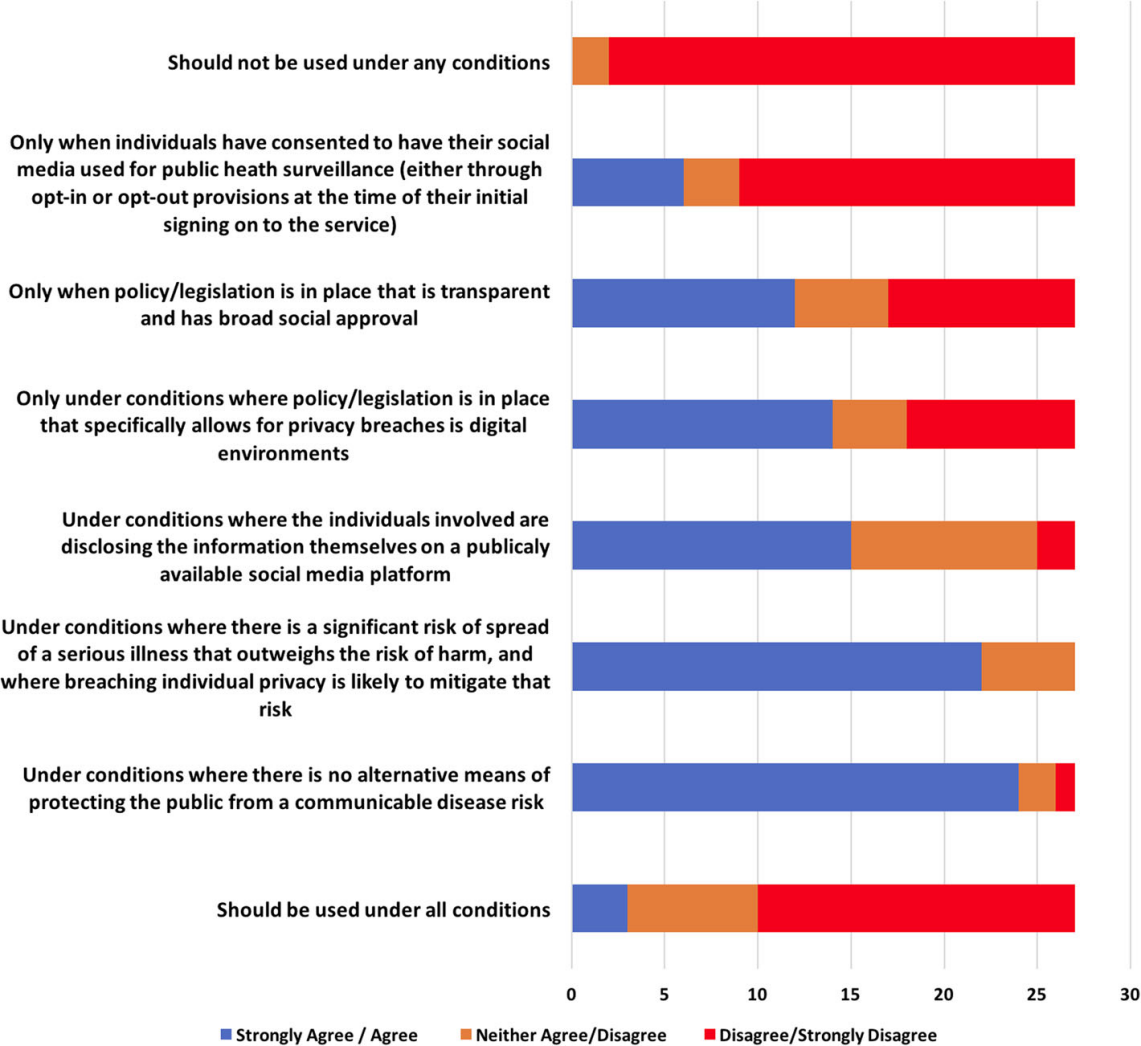
Panellist’s preferences for the appropriate use of social media for event-based enhanced epidemiological surveillance (Round 3)

When asked to answer either yes or no, 83% of round 2 Delphi panellists agreed that members of the public should expect that their activities on social media could be used for public health surveillance. Using a list drawn from responses to previous rounds, panellists were asked, in round 3, to indicate the conditions under which they believed surveillance of social media is appropriate (Fig. 2). The key differences (revealed in the comments) were the extent to which participants were concerned about the lack of a clear legal and social mandate and the potential for negative public reactions. Many panellists also expressed doubts about the veracity and validity of data passively collected from online sources:

DP #37: “*…we don’t know how reliable social media actually is in these circumstances. People post things on social media for lots of reasons. It may not be accurate and could be misleading. So use of the data may lead to a wasteful, inappropriate and/or damaging response.*”

Taken together, these results indicate that most experts agree that using social media as a source of data for routine population health surveillance does not raise significant ethical concerns. However, there is considerable uncertainty about the legitimacy and acceptability of moving beyond population surveillance to use these online systems as a platform for targeted interventions during a public health response, especially when the individuals involved might be publicly identifiable. The sectoral affiliation of panel participants did not appear to influence responses, except in the case of those with legal backgrounds, who strongly preferred that any of the activities discussed should take place under the auspices of a dedicated legislative framework.

### Enhanced communicable disease surveillance in hospital/workplace environments

The second scenario described the use of a novel, highly discriminatory WGS strain-typing system that can routinely type all methicillin-resistant *Staphylococcus aureus* isolates from hospital patients. Because WGS technology can provide a pathogen’s unique genetic ‘fingerprint’, it can potentially identify the timing and direction of individual person-to-person transmission events. In the scenario, the new technology shows that two premature babies who were cared for and died in the same neonatal intensive care unit both had the same rare community-associated methicillin-resistant *Staphylococcus aureus* strain.

Panellists agreed that the most important measures to be taken in response to this scenario were a thorough cleaning of the neonatal intensive care unit facility and a renewed focus on infection control. There was, however, disagreement as to how WGS and strain-typing technologies should be used to mitigate the risks of further infections (other infants, parents, staff, etc.). Most prominent were varying levels of concern about (1) the lack of consent from the parents to use WGS on the isolates collected from the dead neonates for the purposes of conducting an epidemiological investigation; (2) the confidentiality of information elicited through testing and strain typing isolates taken from other patients, parents and/or staff members; and (3) the impact on a staff member or parent if they were found to be the source of the pathogen.

Nevertheless, panellists saw an overriding need to address the outbreak in the interests of public safety, irrespective of any broader questions about consent, confidentiality and discrimination. As one panellist noted:

DP #07 “*…the main issue, is not whether strain typing is done, but how the results are handled. It is in everyone’s interest to understand how pathogens are transmitted so that transmission can be prevented in future, but there should never be any blame (or, hopefully guilt) or penalties involved.*”

**Fig. 3:**
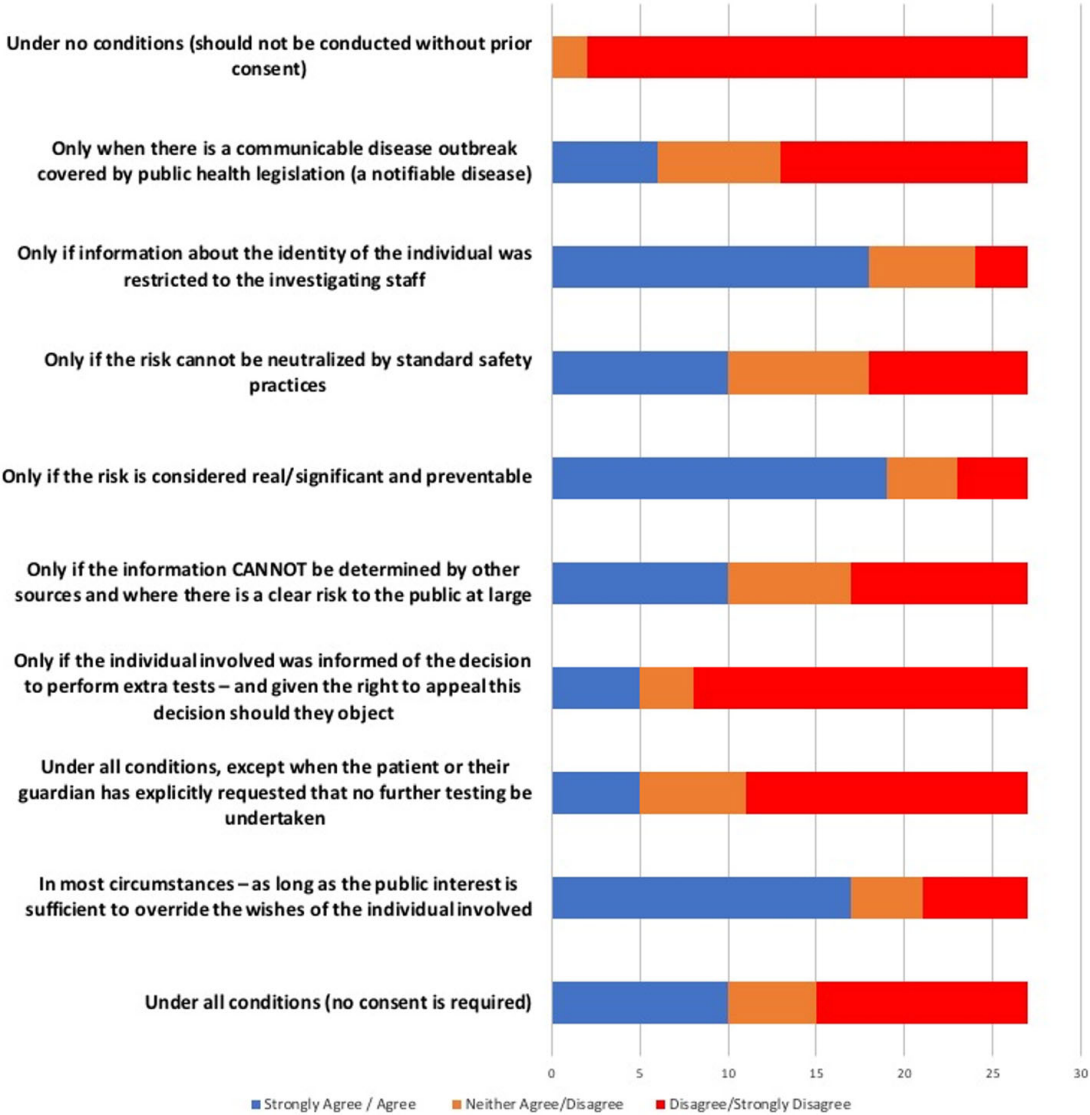
Panellist’s preferences as to the conditions under which the possession of isolates collected from individuals should carry with it the authority to conduct a test that was not agreed to at the time of collection (Round 3)

To explore this issue further, panellists were asked, in round 3, to indicate under what conditions they believed it is appropriate to waive consent from individuals (patients, families and/or staff) and perform a test for an epidemiological investigation aimed at addressing a potential risk to the health of future patients. Figure 3 shows that most panellists held that the protection of the public was of overriding importance and that most of the potential harms could be managed by establishing systems to maintain the confidentiality of those effected. However, differences in the priority afforded by panellists to different conditions of use, such as absence of alternatives, rights of appeal, and likely effectiveness, indicate that the appropriate thresholds for taking such actions remained contentious. Once again, neither the policy network from which participants were recruited, nor their sectorial background appears to have been a major influence on panellists’ opinions and perspectives.

### Enhanced communicable disease surveillance in commercial environments

The final scenario described a significant and fatal outbreak of infection due to a rare strain of *Listeria* (15 cases/5 deaths) in the community, which appears to be linked to the consumption of chicken-wraps produced by a commercial food company. In this scenario, a dispute arises between public health authorities, one of the victim’s families and the food company as to the limits of ‘commercial-in-confidence’ and who controls access to isolates (and any related information) previously submitted voluntarily by food producers to commercial food laboratories for routine fee-for-service safety testing.

Panellists’ responses to this scenario focused on the need to establish who was the legal owner of the isolates held by the commercial food laboratory and whether private companies have the right to block further testing (WGS or otherwise) that lies outside the purpose for which samples were submitted. New technologies such as WGS were seen as being valuable to the investigation of food-borne diseases. However, they also increase commercial/reputational risk for companies who routinely test their products, such that:

DP #22 “*…mandated access* [to commercial samples and test results] *may result in changes to testing in the commercial domain (e.g. cessation of some testing) such that isolates are no longer available… The relationships between labs, food businesses and primary industry and public health agencies are important, as are the priorities of each entity.*”

In round 2, panel members were split as to whether WGS testing should proceed without the permission of the food company (12 in favour, 10 against, 9 uncertain). In their comments, many panellists were keen to emphasise that public interest should always trump commercial interest, and questioned how a company could have effective property rights over a pathogen contaminating its product. To explore this issue further, panellists were asked, in round 3, to indicate the conditions under which they believed it was appropriate to conduct a test on an isolate collected from a commercial company without prior consent.

**Fig. 4:**
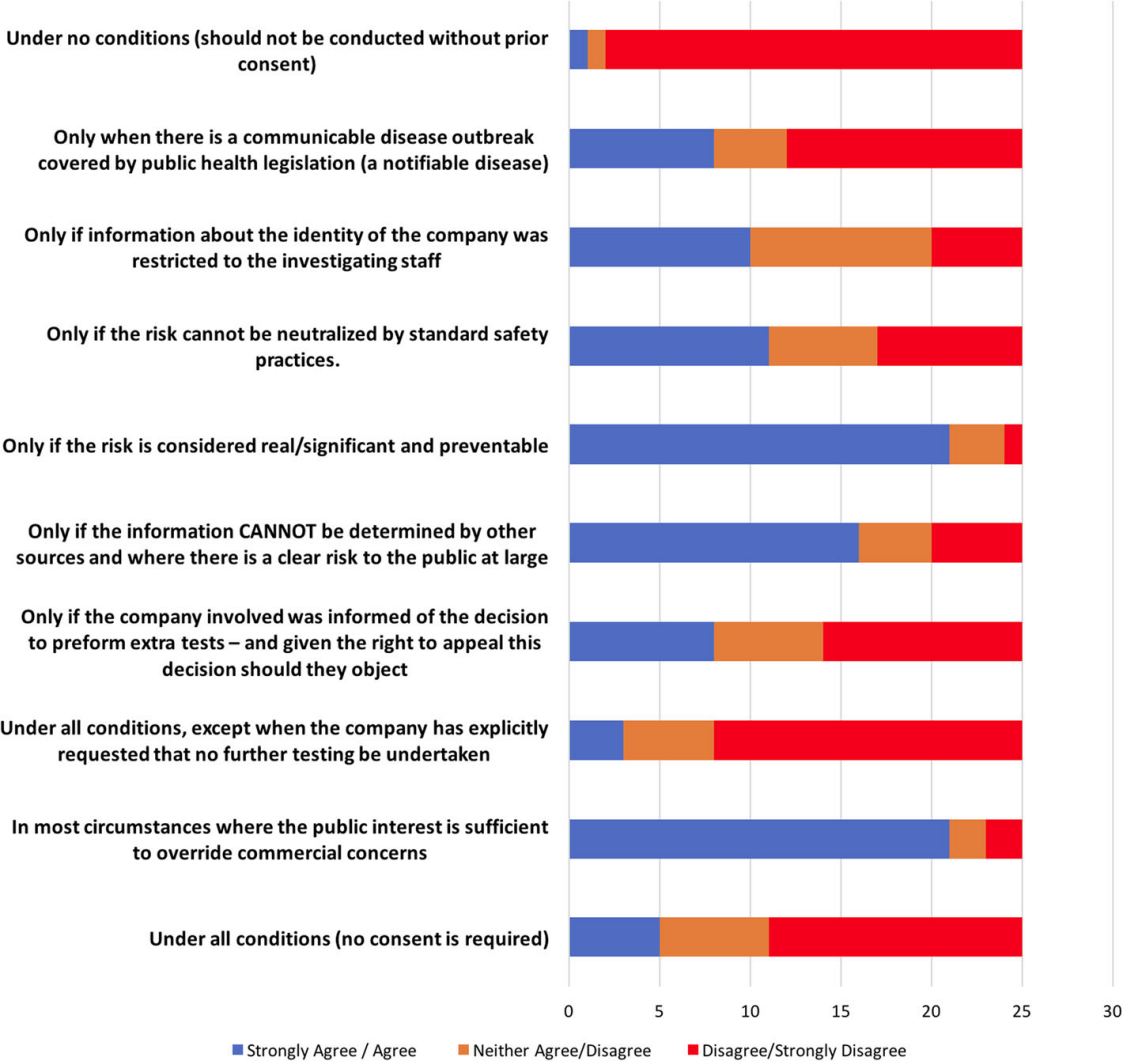
Panellist’s preferences as to the conditions under which the possession of isolates collected from a commercial company should carry with it the authority to conduct a test not agreed to at the time of collection (Round 3)

Figure 4 indicates that significant disagreement remained as to what conditions were required for testing to be appropriate. More detailed analysis of individual responses revealed that these differences were also present within disciplinary groups and across policy networks. Reflecting on their experiences, many, but not all, panellists from the communicable disease policy network wanted to establish a clear procedural separation; they wanted to distinguish the need to be able to perform secondary tests on isolates submitted to commercial food laboratories in order to inform epidemiological investigations (finding out what is going on) from issues of commercial-in-confidence and reputational damage (managing commercial and property issues raised by the public health response), whereas participants from both networks with legal backgrounds wanted matters of ownership and access to information to be resolved before any testing could take place. To provide remedies to this situation (in the longer term) participants suggested that legal clarity on the issue of public health access to commercial data and environmental and food isolates needed to be established.

### Participant perceptions of the risks of technologically enhanced communicable disease surveillance

The application of new technologies to communicable disease surveillance can provide great benefits but also create risks. In round 3, we asked participants to rank the four most significant and four most likely risks for individuals (11 items) and for the broader community (9 items) from lists compiled from previous survey rounds. Final rankings were determined by assigning a score equivalent to reverse rank (e.g. a score of 4 to items rated 1st, 3 for items ranked 2nd, … 1 for items ranked 4th); scores were multiplied by the number of participants who gave each rank and the overall ranking was determined by adding scores for each item. Additional file 3: Tables S2 and S3 respectively show the final rankings the panel gave to risks for individuals and risks for the community.

For individuals, participants ranked the psychological consequences of knowing one’s social media posts are being monitored and the risk of breach in data security highest, with these unwanted outcomes being seen as both significant and likely (Additional file 3: Table S2). Instances and potential social consequences of breaches in the privacy of individuals from primary or secondary data usages such as loss of privacy or stigma were not seen as being major risks. In their comments, many panellists noted that consent for data linkage and secondary use was implied, even as they shared concerns that an erosion of community trust in health authorities was the most significant risk from the systematic use of these technologies (Additional file 3: Table S3). Notably, the veracity of the data produced by enhanced surveillance techniques was once again a key concern among panel members, with the risk to the community of unnecessary fear-mongering and resource misallocation (through individuals changing their behaviour) being ranked as significant and likely.

## Discussion

Participants in this Delphi study, whom we assumed to be representative of policy-makers and other stakeholders involved in decision-making about new technologies for communicable disease research and surveillance, shared a belief in their potential benefits. However, our findings indicate that both groups were uncertain about the public acceptability of routine collection and linkage of such data to enhance surveillance. Despite broad agreement that it could be ethically justified, participants were uneasy about creating a perception of threat to individual rights, which could erode public trust. The panel identified the key challenge to systematic use of new technologies as a need to establish publicly supported guidelines for their operation.

There are good reasons for participants’ hesitation about the use of these technologies, without a clear social licence. The socio-political impacts of technological innovation and emerging risk uncertainty can quickly undermine public trust in governments and, by implication, the authority and perceived legitimacy of associated policy communities – as events surrounding the introduction of mobile telephone masts and genetically modified crops attest [36, 37]. More broadly, political sensitivities are easily heightened as new surveillance systems are introduced – concerns about privacy, consent and other individualistic values tend to dominate public discourse [38]. In practice, however, when compared to other state-led surveillance practices, typically there has been little public opposition to use of personal information by health authorities for the purposes of protecting public health [8, 39]. In recent decades, for the reasons described above, opposition to the use of health specific surveillance data in new ways has been more likely among public health officials than members of the wider public [40–42]. Participants of this Delphi were strongly in favour of enhancing communicable disease surveillance, with many supporting a need for greater public interest consideration, not more privacy.

Where the panellists failed to agree was around appropriate thresholds for public health action and policy protections to be offered to subjects of enhanced surveillance systems. For example, there was consistent disagreement, across all three survey rounds, about the relative importance of confidentiality and public health risk and prioritisation of reasonable alternative interventions; these differences need attention and resolution. Experience shows that, with current analytical tools, interpretation of passively collected and analysed online or pathogen WGS data raises questions about what counts as significant or compelling evidence and what action, if any, should follow [43]. The absence of a policy framework to provide institutional support for new surveillance systems and guide subsequent public health actions comes at a cost [4]. Surveillance systems have limited effectiveness unless they lead to an organised response [44]. Although impossible to quantify, systematic non-use of data can amplify and compound the harms associated with inaction [22]. For reasons of transparency and public acceptability, if not protection, resolving these issues should be a priority because not collecting and using data of significance to public health can lead to preventable harms [8, 45].

Against this background, the relevant Australian legislation prioritises the pursuit of public goods, such as effective communicable disease surveillance, over protecting private interests. Neither individuals nor companies have a broad right to privacy under Australian law. Instead, the law in this area balances public interest against protecting individual or commercial privacy interests and includes a range of protections for the public [46]. This extends to cyber law, which operates under a general principle that social media users own the online content they create, but have limited rights to control its secondary use by others (either individuals or organisations). Similarly, it is a long-established principle under Australian common law that there is no property in human biological samples. This means a person does not own biological samples taken from them (tissues or microbial isolates), nor does the laboratory or facility that holds the samples. As long as there is a clear public health (rather than research) purpose for epidemiological investigation, it follows that there are no significant legal barriers preventing laboratories from performing secondary tests on isolates without the permission of the individuals from whom they were collected [47].

In contrast, it would appear that food or environmental samples (including those collected from livestock) are considered property under the law, such that the Public Health and Food Acts do not clearly establish that further testing of the isolate is legal without strong evidence that the threat to public health is ongoing [48]. Most members of the panel were perplexed by this impediment to secondary testing and argued that public interests should always override any property claims. We note that the case for such a pragmatic approach to issues of consent and ownership for secondary testing of isolates owned by commercial entities is bolstered by epidemiological considerations. Some degree of strain typing must be done routinely if this practice is to be useful for identification of transmission events [5, 16]. Therefore, legal reform may be required because any insistence on a high threshold of public health risk before pathogen WGS can be done would limit the timeliness and, consequently, the effectiveness of this type of communicable disease surveillance. The development of clear guidelines for the secondary testing of patient, food and environmental isolates should be a priority before the use of WGS in epidemiological investigations becomes routine.

Finally, our study reveals a gap between existing policy structures relevant to the use of WGS technologies and social media monitoring for public health purposes, and the individualistic focus of the concerns of most participants. As is the case with many activities undertaken in pursuit of public health, some form of trade-off between public and private interests is necessary. In order to strike the right balance, it is important that the potential benefits and harms of enhanced communicable disease research and surveillance are weighed appropriately. The content of interactions between participants from the different policy networks sampled demonstrated a range of attitudes as to how health authorities should collect, link and use data. The panel concluded that privacy concerns and potential conflicts with commercial interests caused by enhanced surveillance techniques need to be managed appropriately, but neither should substantively limit the pursuit of public interests as important as effective communicable disease prevention and control. However, efficient surveillance across a large population is not achievable unless most people participate. A structured dialogue between interested groups and development of an ethically and legally defensible rationale for the design and operation of surveillance systems [49], prior to their implementation, will help to reassure the public and other stakeholders and enhance the likely success of these systems.

### Strengths and limitations

Delphi survey methods have several well-known limitations, including that the substantive outcomes consist of a set of group intuitions and perspectives, constructed through highly structured social processes of justification among experts [50]. However, as noted in the introduction, the method is particularly useful for the evaluation of complex problems where the rate of social or technological change exceeds the rate of innovation in governance, where evidence that can adequately explain or predict social actions is lacking, or both [25]. The initial response to participant invitations for the current survey was sufficient to generate a lively debate, which was gratifying, given that our invitation was unsolicited. Retention of participants over successive rounds was moderate and the balance between members of different sectors remained constant. Moreover, allowing participants to express their views and comment on each other’s interpretation, via open-ended free text questions, over multiple survey rounds increased the reliability of the study and improved the robustness of the results.

This Delphi survey has captured the perspectives of representatives of expert groups concerned with enhancing communicable disease surveillance systems in Australia. Because of differences in cultural norms and the surrounding social, legal and public health systems, a similar group brought together in another jurisdiction may come to different conclusions. Perspectives of the Australian public are currently being sought through a series of citizens’ juries, to explore what they believe to be acceptable and legitimate use of Big Data and pathogen WGS for the purposes of communicable disease surveillance [51, 52].

## Conclusion

The results of this Delphi survey suggest that there is broad support for using event-based social media monitoring and pathogen WGS technologies to enhance communicable disease surveillance systems across sectoral groups and relevant policy networks. Panellists agreed there is a need to establish a policy framework to ensure appropriate safeguards are in place to protect privacy and that the public is consulted so that they are not unnecessarily alarmed by, or suspicious of, the introduction of new processes for data collection and analyses. However, the emphasis of reforms should be on enabling effective research and surveillance to be conducted, where common benefits are possible. In this regard, the concerns of Australian policy-makers and experts on this issue are not unique. A recent WHO review points to the need to engage with affected communities to establish the conditions and protections under which it is acceptable for surveillance to take place and develop institutional mechanisms that ensure ethical issues are systematically addressed before data collection, use and dissemination [45]. Given that these technologies are already available and have the potential to enhance the capacity of Australian and other health authorities to investigate and prevent outbreaks of infectious disease, with their attendant social and economic costs, the development of clear ethical and legal guidance is urgently needed. The absence of such policy and procedural protections means that public health authorities are likely to only employ these new technologies sporadically, such that opportunities to protect individuals and the wider population from harm will be missed.

## Additional files

Additional file 1 The three hypothetical scenarios describing the use of a new technology for the purposes of communicable disease surveillance in (1) social media/online, (2) hospital/workplace, or (3) commercial environments. (DOCX 10786 kb)

Additional file 2: **Table S1.** Participant characteristics. (DOCX 15 kb)

Additional file 3: **Table S2.** Risks to individuals from enhanced communicable disease surveillance (Round 3). **Table S3.** Risks to the community from enhanced communicable disease surveillance (Round 3). (DOCX 19 kb)

## Abbreviations

WGS: whole genome sequencing
