# Supplementary figures and images for: Perspectives of Australian policy-makers on the potential benefits and risks of technologically enhanced communicable disease surveillance – a modified Delphi survey
Source: Health Res Policy Syst. 2019 Apr 4;17:35. doi: 10.1186/s12961-019-0440-3 (PMC6449976; doi:10.1186/s12961-019-0440-3)

The Round 1 Scenarios


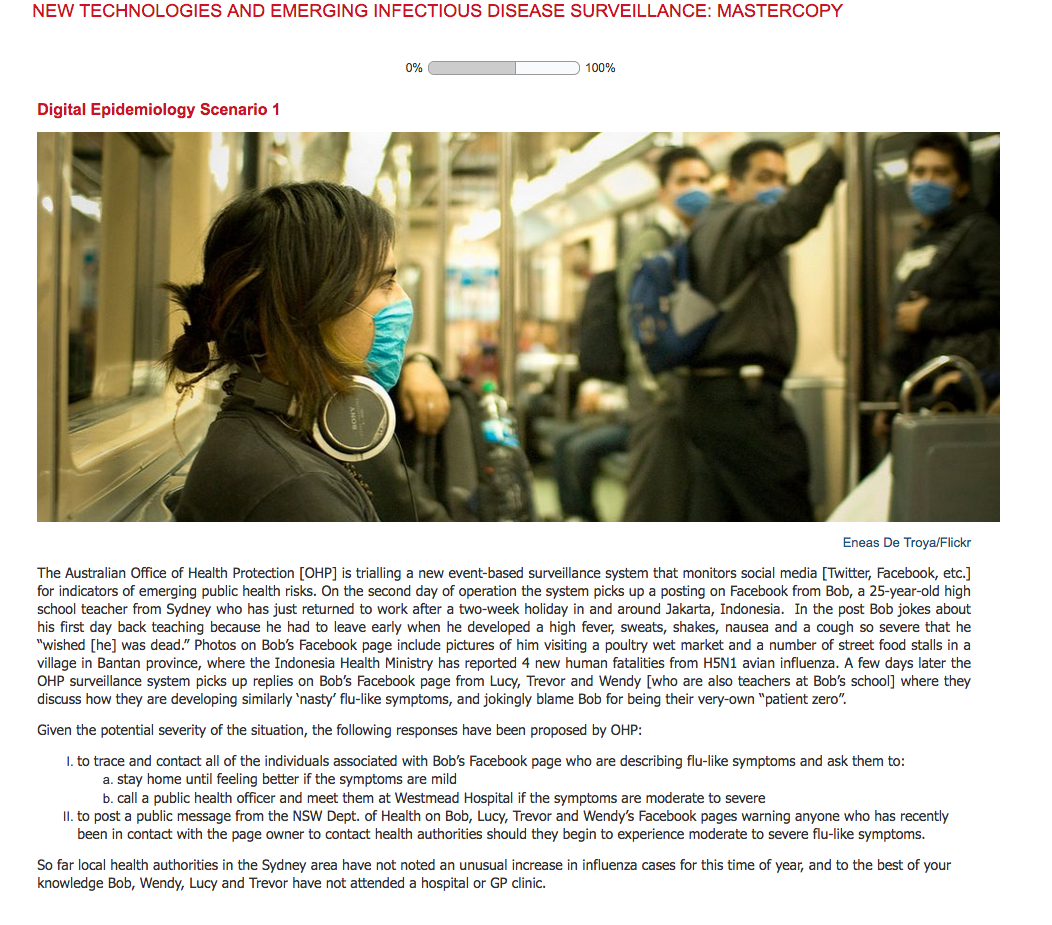


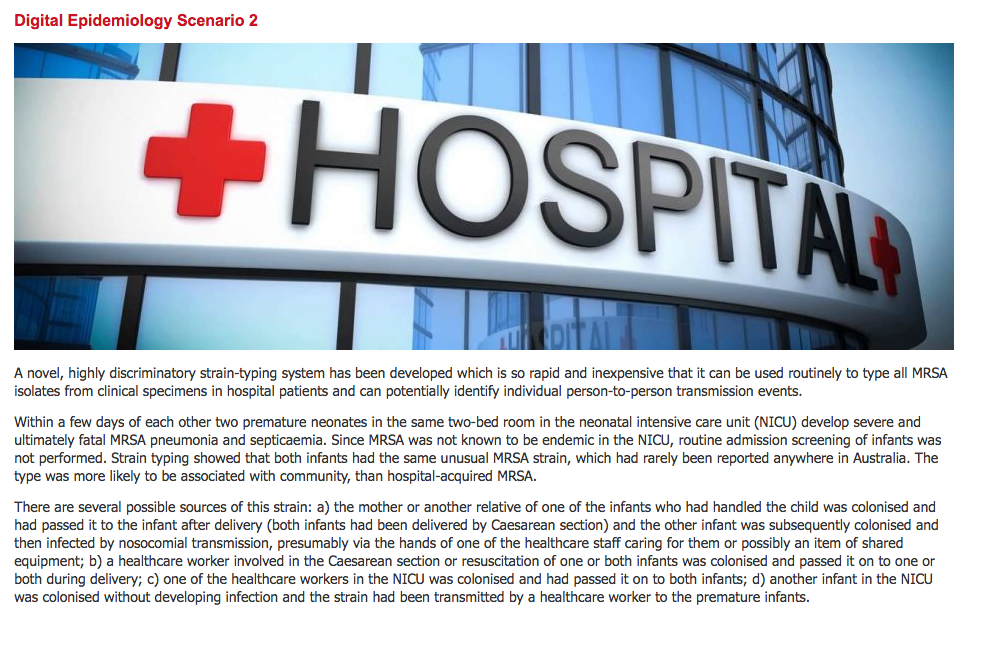

Supplement: Supplementary file 1 — The three hypothetical scenarios describing the use of a new technology for the purposes of communicable disease surveillance in (1) social media/online, (2) hospital/workplace, or (3) commercial environments. (DOCX 10786 kb) [file 12961_2019_440_MOESM1_ESM.docx]
