# Supplementary material for: Perspectives of Australian policy-makers on the potential benefits and risks of technologically enhanced communicable disease surveillance – a modified Delphi survey
Source: Health Res Policy Syst. 2019 Apr 4;17:35. doi: 10.1186/s12961-019-0440-3 (PMC6449976; doi:10.1186/s12961-019-0440-3)
Supplement: Supplementary file 2 — Table S1. Participant characteristics. (DOCX 15 kb) [file 12961_2019_440_MOESM2_ESM.docx]

| **Table S1: Participant Characteristics** | | | |
| --- | --- | --- | --- |
|  | **Round 1** | **Round 2** | **Round 3** |
|  | n=44 | n=31 | n=27 |
| Response rate | 47% | 71% | 87% |
| **Employment setting** |  |  |  |
| Federal Government | 2 (0.045)* | 1 (0.032) | 1 (0.037) |
| Provincial Governments | 8 (0.205) | 8 (0.258) | 8 (0.296) |
| Health Agency / Industry | 11 (0.25) | 8 (0.258) | 6 (0.222) |
| University / NGO | 23 (0.523) | 14 (0.452) | 12 (0.444) |
|  |  |  |  |
| **Primary role / responsibility** |  |  |  |
| Current / Former Chief Medical Officers | 3 (0.068) | 2 (0.065) | 2 (0.074) |
| Directors of Health / Security Agencies | 19 (0.431) | 15 (0.484) | 13 (0.481) |
| Medical or Legal Academic / Practitioner | 22 (0.5) | 14 (0.452) | 12 (0.444) |
|  |  |  |  |
| **Disciplinary / sectoral background** |  |  |  |
| Communicable Disease Control | 30 (0.682) | 23 (0.741) | 20 (0.741) |
| Health, Commercial or Technology Law | 14 (0.318) | 8 (0.258) | 7 (0.259) |
| * Data in brackets are proportions of total in each category | | | |
