# Supplementary material for: Perspectives of Australian policy-makers on the potential benefits and risks of technologically enhanced communicable disease surveillance – a modified Delphi survey
Source: Health Res Policy Syst. 2019 Apr 4;17:35. doi: 10.1186/s12961-019-0440-3 (PMC6449976; doi:10.1186/s12961-019-0440-3)
Supplement: Supplementary file 3 — Table S2. Risks to individuals from enhanced communicable disease surveillance (Round 3). Table S3. Risks to the community from enhanced communicable disease surveillance (Round 3). (DOCX 19 kb) [file 12961_2019_440_MOESM3_ESM.docx]

| **Table S2: Risks to Individuals from Enhanced Communicable Disease Surveillance (R3)** | | | | |
| --- | --- | --- | --- | --- |
|  | **Most Significant Risks** | | **Most Likely Risks** | |
|  | **Overall ranking** | **Ranking score** | **Overall ranking** | **Ranking score** |
| Individuals experiencing insecurity and fear about their personal data being collected and stored | 1 | 52 | 1 | 52 |
| Data insecurity / inadequate protection of screening information | 2 | 46 | 2 | 44 |
| Loss of confidence in public health authorities & Australian healthcare system more generally | 3 | 35 | 6 | 23 |
| Individuals experiencing ostracism, guilt and stigma based on outcomes of secondary data uses | 4 | 25 | 7 | 14 |
| Loss of privacy and confidentiality | 5 | 24 | 3 | 34 |
| Government linking this with other information. eg. to Centrelink | 6 | 15 | 8 | 10 |
| Loss of ability to earn a living | 7 | 14 | 11 | 0 |
| Diminished access to health care resources for people identified as non-symptomatic disease carriers | 8 | 13 | 9 | 9 |
| Lack of consent - including the use of data/samples for tests other than those originally intended | 9 | 8 | 4 | 27 |
| Requiring individuals to undergo testing that do not provide them with therapeutic benefits | 10 | 6 | 5 | 31 |
| Loss of social status | 11 | 2 | 10 | 2 |

| **Table S3: Risks to the Community from Enhanced Communicable Disease Surveillance (R3)** | | | | |
| --- | --- | --- | --- | --- |
|  | **Most Significant Risks** | | **Most Likely Risks** | |
|  | **Overall ranking** | **Ranking score** | **Overall ranking** | **Ranking score** |
| Erosion of trust in health authorities due to invasive use of technology | 1 | 50 | 6 | 22 |
| Un-validated data directing finite public health resources | 2 | 42 | 2 | 39 |
| The potential for public fear to spread quickly | 3 | 42 | 3 | 34 |
| Lack of transparency (in the absence of a policy framework developed ex ante) | 4 | 34 | 4 | 33 |
| Reliance upon for profit companies who may not comply standards of ethical and legal conduct | 5 | 29 | 5 | 29 |
| Potential for manipulation of data through individuals changing their behavior/what they report online | 6 | 28 | 1 | 42 |
| Economic risk (companies going bankrupt) from un/warranted reputational damage | 7 | 21 | 8 | 17 |
| Potential for manipulation of data through prank or malicious activities by members of the public | 8 | 18 | 7 | 21 |
| International users adding irrelevant data to social media platforms | 9 | 0 | 9 | 3 |
